# The gut microbiota and plasma metabolites on functional dyspepsia: a study integrating Mendelian randomization and experimental validation

**DOI:** 10.3389/fmed.2026.1793831

**Published:** 2026-03-16

**Authors:** Xinxin Hu, Xueping Zhang, Chen Yang, Lei Chen, Ruirong Yang, Chengxiang Wang, Suowei Wu, Lixin Ma, Wenqi Jiang, Pumin Deng, Xiaolan Su, Wei Wei

**Affiliations:** 1 Department of Gastroenterology, Wangjing Hospital, China Academy of Chinese Medical Sciences, Beijing, China; 2 Department of Gastroenterology, Emergency General Hospital, Beijing, China; 3 Graduate School, Beijing University of Chinese Medicine, Beijing, China

**Keywords:** alpha-hydroxyisocaproate, functional dyspepsia, kynurenate, Mendelian randomization, *p_ Actinobacteria*

## Abstract

**Objective:**

This two-sample Mendelian randomization (MR) investigation aimed to explore how gut microbiota influences functional dyspepsia (FD) via plasma metabolites. The results were then confirmed *in vivo*.

**Methods:**

A GWAS of 7,738 people was used to generate genetic instruments for 412 gut microbiota characteristics. The NHGRI-EBI GWAS Catalog listed 1,400 plasma metabolites, and the FinnGen biobank provided FD summary statistics. Using the inverse-variance weighted (IVW) approach, potential causal links between gut microbiota and plasma metabolites in FD were assessed. To make sure it was resilient, pleiotropy and heterogeneity evaluations were performed. For experimental validation, the combination of 0.2% iodoacetamide gavage combined with tail-clamp stress was used to create an FD rat model. Fecal and plasma samples from the rats were analyzed to verify the MR findings.

**Results:**

Three gut microbiota taxa and two microbial metabolic pathways were identified to be associated with FD. The superpathway of pyridoxal 5′-phosphate biosynthesis and salvage (OR: 0.89; 95% CI: 0.81–0.97; *p* = 0.01), *p_Actinobacteria* (0.85, 0.77–0.94, *p* = 0.002), and *s_Bifidobacterium adolescentis* (0.86, 0.75–0.97, *p* = 0.017) were protective factors against FD. In contrast, the superpathway of menaquinol-8 biosynthesis II (1.13, 1.03–1.25, *p* = 0.01) and *s_Lachnospiraceae bacterium_5_1_63FAA* (1.09, 1.02–1.16, *p* = 0.008) were identified as risk factors. MR analysis of 1,400 plasma metabolites using IVW, weighted median, and other methods (*p* < 0.05), along with pleiotropy and heterogeneity tests, suggested that these three microbiota features and two pathways may influence FD pathogenesis through the regulation of 81 plasma metabolites or metabolite ratios. Animal studies further identified the *p_ Actinobacteria* and two differential metabolites: alpha-hydroxyisocaproate and kynurenate.

**Conclusion:**

Three gut microbiota taxa and two pathways reflecting microbial composition and activity were associated with FD and linked to alterations in 81 metabolites or metabolite ratios. This study identifies a correlation between altered *p_ Actinobacteria* abundance and changes in alpha-hydroxyisocaproate and kynurenate levels in FD, offering a perspective centered on the gut microbiota and metabolites for the early detection, diagnosis, and treatment of this condition.

## 1 Introduction

In the absence of any organic condition that could account for the symptoms, functional dyspepsia (FD) is a clinically prevalent functional gastrointestinal ailment marked by premature satiety, epigastric pain, burning, and postprandial fullness (1). Although the exact pathophysiology of FD is yet unknown, it is thought to involve disorder of the brain-gut axis, visceral hypersensitivity, and gastrointestinal motility abnormalities. Its global prevalence is between 8 and 12%, and it is rising yearly (2). Recently, the theory of the “brain - gut - enteric microbiota axis” has emphasized the crucial role of gut microbiota in FD (3, 4). Dysbiotic microbiota contribute to the course of FD by influencing intestinal immunological and inflammatory responses, interfering with vagal nerve communication, and playing a role in mood control and central nervous system modulation (5–7). Therefore, targeting the gut microbiota and its metabolites represents a promising therapeutic strategy for FD. Nevertheless, owing to the high complexity of the human gut microbiome, identifying causal microbial and metabolic contributors to FD remains a considerable challenge.

**Figure 1:**
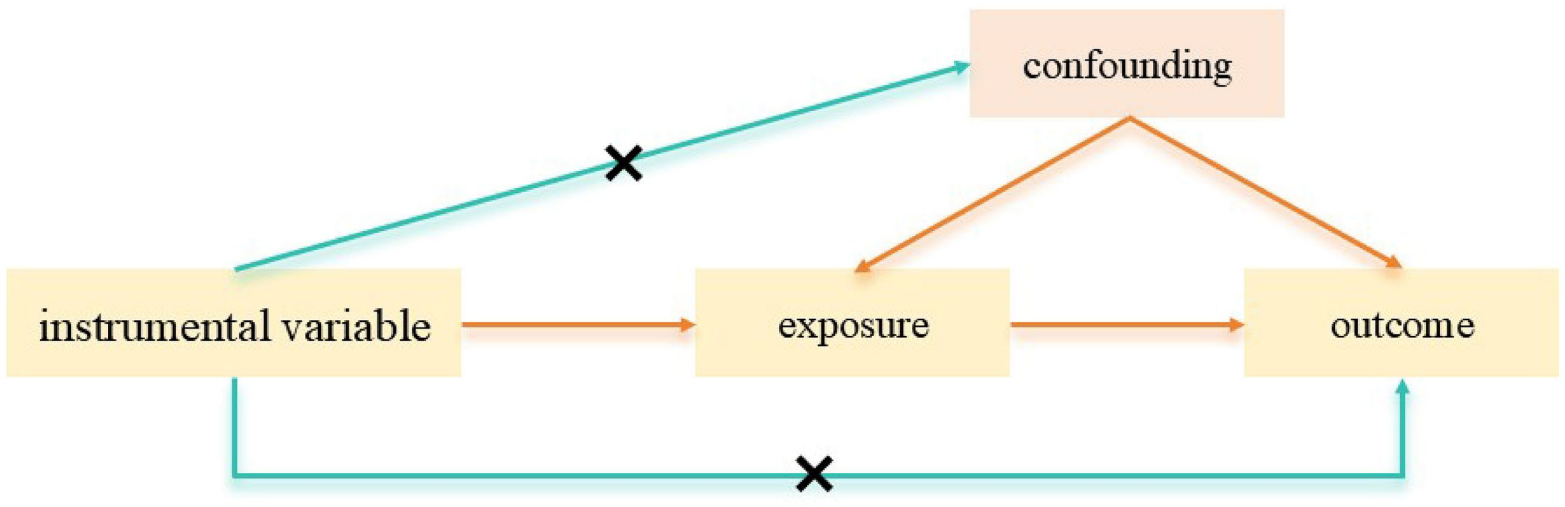
Hypothesis of Mendelian randomization.

Based on the principles of Mendel’s second law (8), Mendelian randomization (MR) employs genetic variants, particularly single nucleotide polymorphisms (SNPs), as instrumental variables (IVs) to deduce possible causal relationships between exposures and outcomes from observed associations (9); Figure 1 illustrates the MR principle schematically. Owing to the stochastic segregation of alleles in meiosis and the relative stability of genetic information across disease progression, MR is often considered a form of RCT, effectively circumventing many constraints typical of traditional observational epidemiology (10). Using a two-sample MR analysis, we investigated the causal relationships between gut microbiota, plasma metabolites, and FD, with further validation via animal experiments to elucidate underlying causal mechanisms.

## 2 Methods

### 2.1 Data sources for exposures and outcomes

Summary-level data for 412 human gut microbiota features were obtained from the Dutch Microbiome Project, which included 7,738 participants and covered 207 taxa (5 phyla, 10 classes, 13 orders, 26 families, 48 genera, 105 species) and 205 pathways reflecting microbial composition and activity (11) (Supplementary Table 1). The Canadian Longitudinal Study on Aging (CLSA) cohort, which included 1,091 blood metabolites and 309 metabolite ratios and included 8,299 adults, provided the data for 1,400 plasma metabolites (12) (Supplementary Table 2). The Genome-wide association study (GWAS) summary statistics for FD were derived from the FinnGen Consortium (Release 11),^1^ including 10,851 cases and 385,082 controls of European ancestry. This study did not need further ethical approval or participant permission because it used publicly available summary data.

### 2.2 Instrumental variable selection

We selected SNPs meeting a suggestive significance threshold of *p* < 1 × 10^−5^ for association with gut microbiota as IVs. Linkage disequilibrium (LD) clumping was applied to ensure independence among SNPs (r^2^ < 0.001, distance threshold = 10,000 kb). The LDlink platform^2^ was used to assess associations between selected SNPs and potential confounding phenotypes; SNPs correlated with known confounders were removed (13). Furthermore, SNPs linked to known risk factors for FD were not included, including mood disorders (1), irritable bowel syndrome (14), gastrointestinal infections, and the use of antibiotics or non-steroidal anti-inflammatory medicines. Finally, to mitigate weak instrument bias, only SNPs with an *F* > 10 were retained (Supplementary Table 3).

### 2.3 Mendelian randomization analysis

We applied five MR analytical methods: the inverse-variance weighted (IVW) method—which served as the main approach—along with the weighted median, weighted mode, simple mode, and MR-Egger regression. To evaluate heterogeneity across the selected SNPs, Cochran’s Q test was performed. In cases of significant heterogeneity (*p* < 0.05), the IVW method was applied under a random-effects framework; otherwise, a fixed-effects model was utilized (15). Horizontal pleiotropy was evaluated via MR-Egger regression, with a non-zero intercept indicating potential pleiotropic bias (16). The Mendelian Randomization Pleiotropy RESidual Sum and Outlier (MR-PRESSO) method was used to detect outlier SNPs that may bias the causal estimates and to evaluate the impact of their removal on the results (17). A leave-one-out sensitivity analysis was performed to assess the robustness of the results by iteratively excluding each SNP and re-estimating the causal effect. We first examined the association of gut microbiota with FD, followed by a reverse MR analysis to assess the potential for reverse causality from FD to the identified microbial taxa. We subsequently examined the links between the selected gut microbiota and plasma metabolites, inferred their potential causal relationships, and validated these findings through animal experiments. Odds ratios (ORs) with 95% confidence intervals (CIs) were used to represent correlations between gut microbiota and FD or plasma metabolites. Using the Benjamini-Höberg approach, several comparisons were corrected. MR analyses and visualization of results were conducted in R version 4.4.1, utilizing packages including “TwoSampleMR,” “MR-PRESSO,” “forestploter,” and “circlize.”

### 2.4 Animal model establishment

Twenty 5-day-old male Sprague–Dawley (SD) rats were purchased from Sibeifu Biotechnology Co., Ltd. and housed at the Institute of Basic Theory for Chinese Medicine, China Academy of Chinese Medical Sciences. The animals were maintained under controlled conditions: temperature of 20–24 °C, relative humidity of 40–50%, with automated air circulation and a 12-h light/dark cycle (lights on at 08:00). Animals were allowed free access to food and water for the entire duration of the investigation. Every experimental technique was authorized by the Ethics Committee of Experimental Animal Center of the Institute of Basic Theory for Chinese Medicine, China Academy of Chinese Medical Sciences (Approval No.: IBTCMCACMS21-2410-02).

After 5 days of acclimatization, rats were divided into two groups: the control (CON, *n* = 10) and the model (MOD, *n* = 10). Starting at 10 days of age, MOD rats received daily oral administration of 0.2 mL of 0.1% iodoacetamide (IA) in 2% sucrose for 6 consecutive days, while CON rats received 0.2 mL of 2% sucrose daily. Thereafter, all animals were raised under standard conditions until 7 weeks of age. At week 7, FD modeling was induced in the MOD group via tail pinch stimulation: the distal one-third of the tail was clamped with surgical forceps for 30 min per session, 4 times daily, for 7 days (until 8 weeks of age) to provoke confrontational and agitated behaviors (18, 19). All animals survived the modeling period.

### 2.5 General condition assessment

The general condition of all animals was observed daily throughout the study, with a focus on mental status, autonomous activity, fur appearance, and growth. At the end of the modeling period, body weight and 24-h food intake were measured.

### 2.6 Animal sample collection

Fecal samples were collected after successful model establishment. Rats were fasted for 12 h with free access to water. A semi-solid paste was prepared by dissolving 10 g of carboxymethyl cellulose sodium, 16 g of skim milk powder, 8 g of corn starch, 8 g of sucrose, and 2 g of activated charcoal in 250 mL of distilled water (20). Each rat was orally administered 3 mL of the semi-solid paste by gavage (21). Fifty minutes after gavage, rats were anesthetized with 3% isoflurane. Following anesthesia, the abdominal cavity was surgically opened, and blood samples were collected from the abdominal aorta and centrifuged to obtain plasma, which was stored at −80 °C for further analysis. Rats were then euthanized by cervical dislocation under deep anesthesia. Subsequently, laparotomy was performed to harvest the stomach and small intestine for the assessment of gastric emptying rate and intestinal propulsion rate. After isolation, the stomach was opened along the greater curvature, gently rinsed with ice-cold saline to remove luminal contents, and blotted dry with filter paper. The gastric tissues were then carefully separated and fixed in 4% paraformaldehyde for subsequent histological analysis.

### 2.7 Gastric emptying rate and intestinal propulsion rate

Following administration of the charcoal-labeled semi-solid nutrient paste and subsequent laparotomy, the stomach and small intestine were carefully excised for quantitative assessment. The stomach was first weighed to obtain the total stomach weight. The gastric contents were then completely removed, and the empty stomach was blotted dry and weighed again to determine the empty stomach weight. The gastric emptying rate was calculated using the following formula: Gastric emptying rate (%) = [1 − (total stomach weight − empty stomach weight)/weight of administered semi-solid paste] × 100%. To evaluate intestinal propulsion, the entire small intestine from the pylorus to the ileocecal junction was excised and gently placed on a flat surface without stretching. The total length of the small intestine and the distance traveled by the charcoal marker from the pylorus to its furthest point were measured. The intestinal propulsion rate was calculated as follows: Intestinal propulsion rate (%) = (distance traveled by charcoal marker/total length of the small intestine) × 100% (22).

### 2.8 HE staining

Paraffin-embedded gastric antrum tissues were sectioned into 4–5 μm thick slices and baked at 60 °C for 30 min. For hematoxylin and eosin (H&E) staining, the sections were first stained with hematoxylin for 5–8 min, followed by counterstaining with eosin for 1–3 min. After staining, the sections were sequentially dehydrated in graded ethanol, cleared in xylene, and mounted with neutral resin. Histopathological changes were examined and documented using a light microscope.

### 2.9 16S rDNA sequencing

From each group, six rats were randomly selected for 16S rDNA sequencing. Using the using the VAMNE Stool/Soil DNA Extraction Kit-BOX2 (Vazyme), genomic DNA was isolated from fecal samples, and its purity and concentration were assessed. The V3–V4 hypervariable regions were amplified using particular primers (341F: 5’-CCTAYGGGRBGCASCAG-3′; 806R: 5’-GGACTACNNGGGTATCTAAT-3′). The PCR products were visualized by 2% agarose gel electrophoresis, pooled at equidensity ratios, and purified using the Qiagen Gel Extraction Kit. Sequencing libraries were generated using the Illumina TruSeq® DNA PCR-Free Sample Preparation Kit and sequenced on the NovaSeq6000 platform to generate paired-end 250 bp reads. Sequences were clustered into operational taxonomic units (OTUs) based on a 97% similarity threshold, followed by alpha and beta diversity analyses. Differentially abundant microbial taxa were identified via linear discriminant analysis effect size (LEfSe).

### 2.10 Untargeted metabolomics

Plasma samples were extracted with acetonitrile–methanol to precipitate proteins and centrifuged at 12,000 rpm for 15 min at 4 °C. The supernatants were collected for untargeted metabolomics analysis. A pooled quality control (QC) sample was prepared by mixing equal aliquots of all supernatants and was analyzed intermittently throughout the analytical run to monitor instrument stability and data reproducibility. LC–MS/MS analysis was performed using a UHPLC system coupled with an Orbitrap Exploris 120 mass spectrometer. Chromatographic separation was conducted on a Waters ACQUITY UPLC BEH Amide column with a mobile phase consisting of ammonium acetate and ammonium hydroxide in water (phase A) and acetonitrile (phase B). Mass spectrometry data were acquired in both positive and negative electrospray ionization modes. Raw data were processed with an in-house R program based on XCMS for peak detection, alignment, and quantification, and metabolite identification was performed using the BiotreeDB database. Principal component analysis (PCA) was performed including QC samples to evaluate analytical stability, while orthogonal partial least squares discriminant analysis (OPLS–DA) was used to assess group discrimination. Differential metabolites were identified based on variable importance in projection (VIP) > 1 and *p* < 0.05.

### 2.11 Statistical methods

Statistical analyses and graphing were performed using GraphPad Prism (version 10.0). A two-tailed *p* < 0.05 was considered statistically significant. All quantitative data are presented as mean ± standard deviation (SD). Normality was assessed using the Shapiro–Wilk test, and homogeneity of variance was evaluated using Levene’s test. When both assumptions were satisfied, comparisons between two groups were performed using an independent samples t-test. Additionally, Spearman correlation analysis was performed to assess the associations between differential microbial taxa and differential metabolites.

## 3 Results

### 3.1 Causal effects between gut microbiota and FD

**Figure 2:**
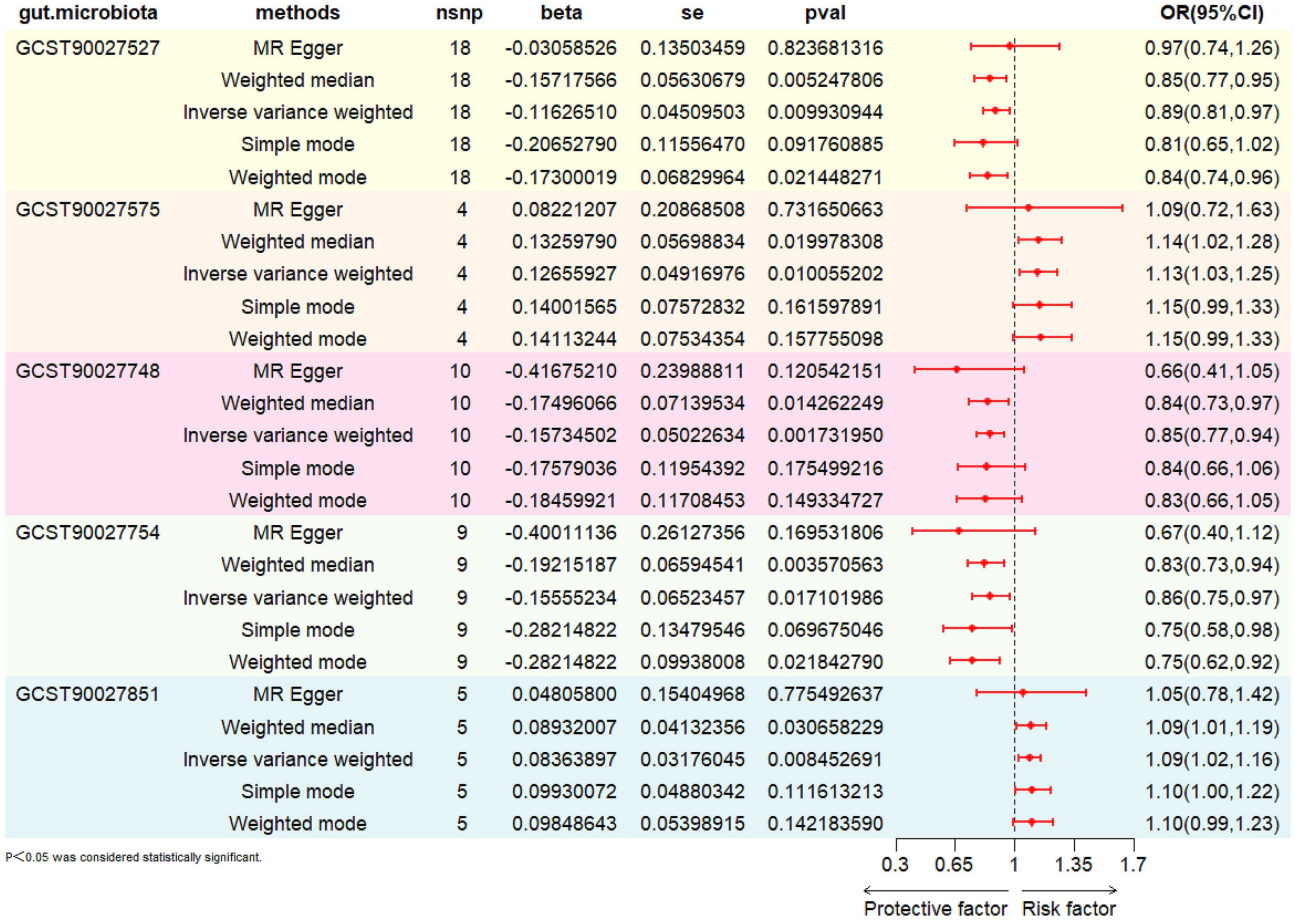
The MR results of the effects of GM on FD.

The associations between 412 gut microbiota with FD are presented in Supplementary Tables 4, 5. Cross-validation using the IVW and weighted median methods identified three gut microbial taxa and two microbial pathways significantly associated with FD (Table 1; Figure 2). The superpathway of pyridoxal 5′-phosphate biosynthesis and salvage (OR: 0.89; 95% CI: 0.81–0.97; *p* = 0.01), the *p_Actinobacteria* (OR: 0.85; 95% CI: 0.77–0.94; *p* = 0.002), and *s_Bifidobacterium_adolescentis* (OR: 0.86; 95% CI: 0.75–0.97; *p* = 0.017) were identified as protective factors against FD. In contrast, the superpathway of menaquinol-8 biosynthesis II (OR: 1.13; 95% CI: 1.03–1.25; *p* = 0.01) and *s_Lachnospiraceae_bacterium_5_1_63FAA* (OR: 1.09; 95% CI: 1.02–1.16; *p* = 0.008) were associated with an increased risk of FD. The leave-one-out sensitivity analysis for gut microbiota on FD is presented in Supplementary Figure 1. Reverse MR analysis did not reveal significant causal effects of FD on gut microbiota (*p* > 0.05, Supplementary Table 6).

**Table 1:** Causal results of gut microbiota on FD.

Gut microbiota	Method	NO. SNP	OR (95%CI)	*p*-value	Heterogenenity		Pleiotropy		MR-PRESSO global test
					Q-value	*p*	Egger intercept	*p*	*p*
GCST90027527	IVW	18	0.89 (0.81–0.97)	0.010	25.96	0.08	/	/	/
	MR Egger		0.97 (0.74–1.26)	0.824	25.24	0.07	−0.01	0.51	0.102
	Weighted median		0.85 (0.77–0.95)	0.005	/	/	/	/	/
GCST90027575	IVW	4	1.13 (1.03–1.25)	0.010	0.15	0.99	/	/	/
	MR Egger		1.09 (0.72–1.63)	0.732	0.10	0.95	0.01	0.85	0.998
	Weighted median		1.14 (1.02–1.28)	0.020	/	/	/	/	/
GCST90027748	IVW	10	0.85 (0.77–0.94)	0.002	8.08	0.53	/	/	/
	MR Egger		0.66 (0.41–1.05)	0.121	6.86	0.55	0.03	0.30	0.506
	Weighted median		0.84 (0.73–0.97)	0.014	/	/	/	/	/
GCST90027754	IVW	9	0.86 (0.75–0.97)	0.017	15.95	0.04	/	/	/
	MR Egger		0.67 (0.40–1.12)	0.170	14.07	0.05	0.03	0.37	0.072
	Weighted median		0.83 (0.73–0.94)	0.004	/	/	/	/	/
GCST90027851	IVW	5	1.09 (1.02–1.16)	0.008	0.80	0.94	/	/	/
	MR Egger		1.05 (0.78–1.42)	0.775	0.75	0.86	0.01	0.83	0.949
	Weighted median		1.09 (1.01–1.19)	0.031	/	/	/	/	/

### 3.2 Causal effects between gut microbiota and plasma metabolites

**Figure 3:**
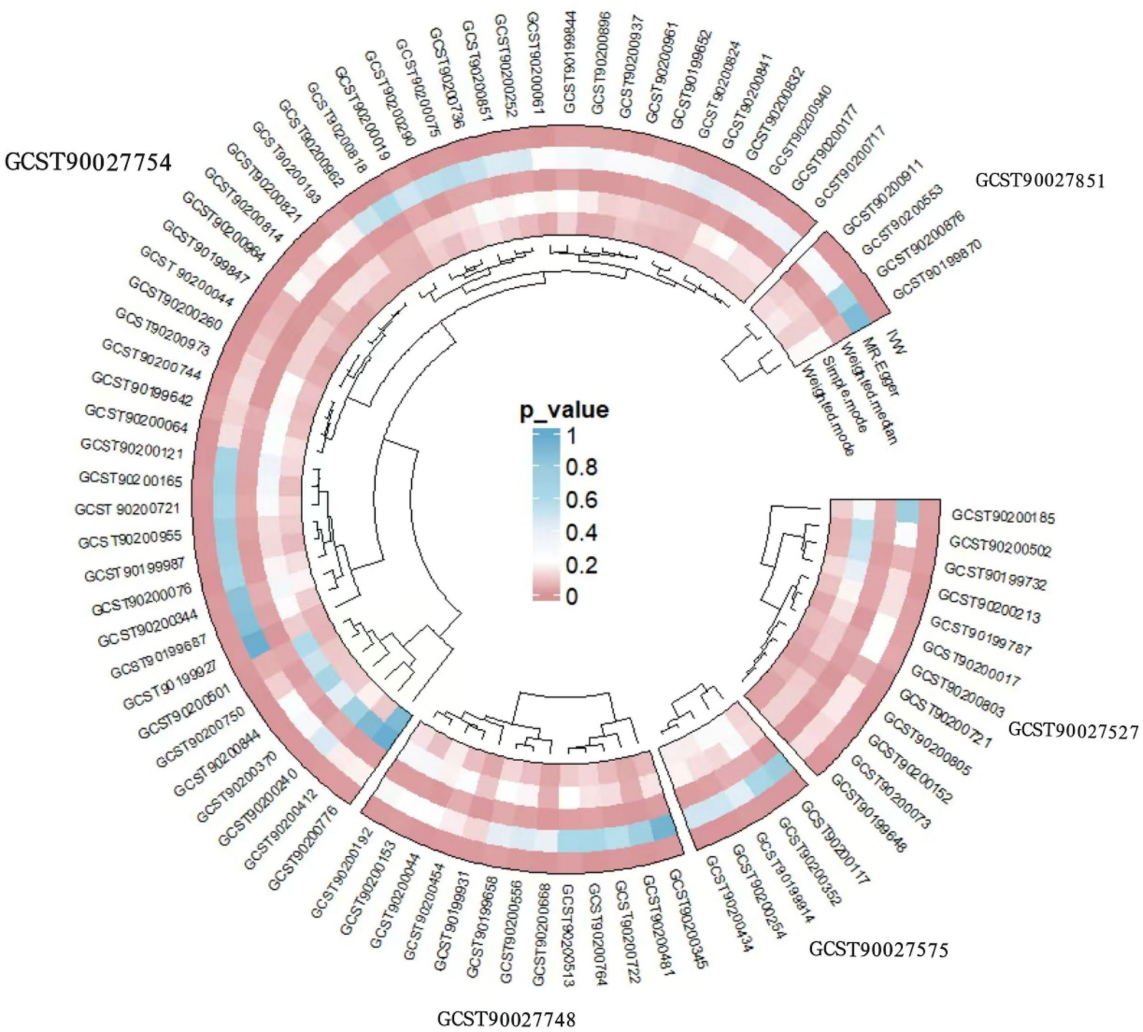
MR results of the effects of five GM taxa on plasma metabolites. The shade of the color reflected the size of the *p*-value within the circle.

The causation between the three gut microbial taxa, two microbial composition and activity pathways, and 1,400 plasma metabolites are summarized in Supplementary Tables 7, 8. Cross-validation using the IVW and weighted median methods identified 81 plasma metabolites and metabolite ratios that were substantially connected to the five candidate gut microbial features. Sensitivity analyses bolstered the robustness of the primary results. We found that the superpathway of pyridoxal 5′-phosphate biosynthesis and salvage exerted a protective effect against FD by modulating 12 metabolites, including adenosine 5′-diphosphate (ADP) to phosphate ratio (OR: 1.18; 95% CI: 1.05–1.32; *p* = 0.006) and 1,5-anhydroglucitol (1,5-AG) (OR: 1.14; 95% CI: 1.04–1.24; *p* = 0.005). Similarly, the *p_Actinobacteria* was inferred to influence FD risk through the regulation of 13 metabolites, such as phosphate to uridine ratio (OR: 1.21; 95% CI: 1.08–1.35; *p* = 0.0008), alpha-hydroxyisocaproate (OR: 0.88; 95% CI: 0.79–0.98; *p* = 0.02), kynurenate (OR: 0.87; 95% CI: 0.78–0.97; *p* = 0.01). *s_Bifidobacterium_adolescentis* was hypothesized to mediate protective effects via 47 metabolites, including 1-(1-enyl-stearoyl)-2-oleoyl-GPE (*p*-18:0/18:1) (OR: 0.76; 95% CI: 0.69–0.84; *p* < 0.0001) and ADP to glycine ratio (OR: 0.76; 95% CI: 0.67–0.88; *p* = 0.0001). Conversely, the superpathway of menaquinol-8 biosynthesis II appeared to promote FD progression by modulating 5 metabolites, such as 12,13-DiHOME (OR: 1.20; 95% CI: 1.07–1.34; *p* = 0.002) and glycosyl-N-(2-hydroxynervonoyl)-sphingosine (d18:1/24:1(2OH)) (OR: 1.17; 95% CI: 1.05–1.31; *p* = 0.003). Similarly, *s_Lachnospiraceae_bacterium_5_1_63FAA* was implicated in FD pathogenesis through the regulation of 4 metabolites, including 4-cholesten-3-one (OR: 1.13; 95% CI: 1.05–1.22; *p* = 0.001) and cortisone to 4-cholesten-3-one ratio (OR: 0.90; 95% CI: 0.84–0.97; *p* = 0.004) (Figure 3). To validate these hypotheses, animal experiments were conducted.

### 3.3 General condition assessment

**Figure 4:**
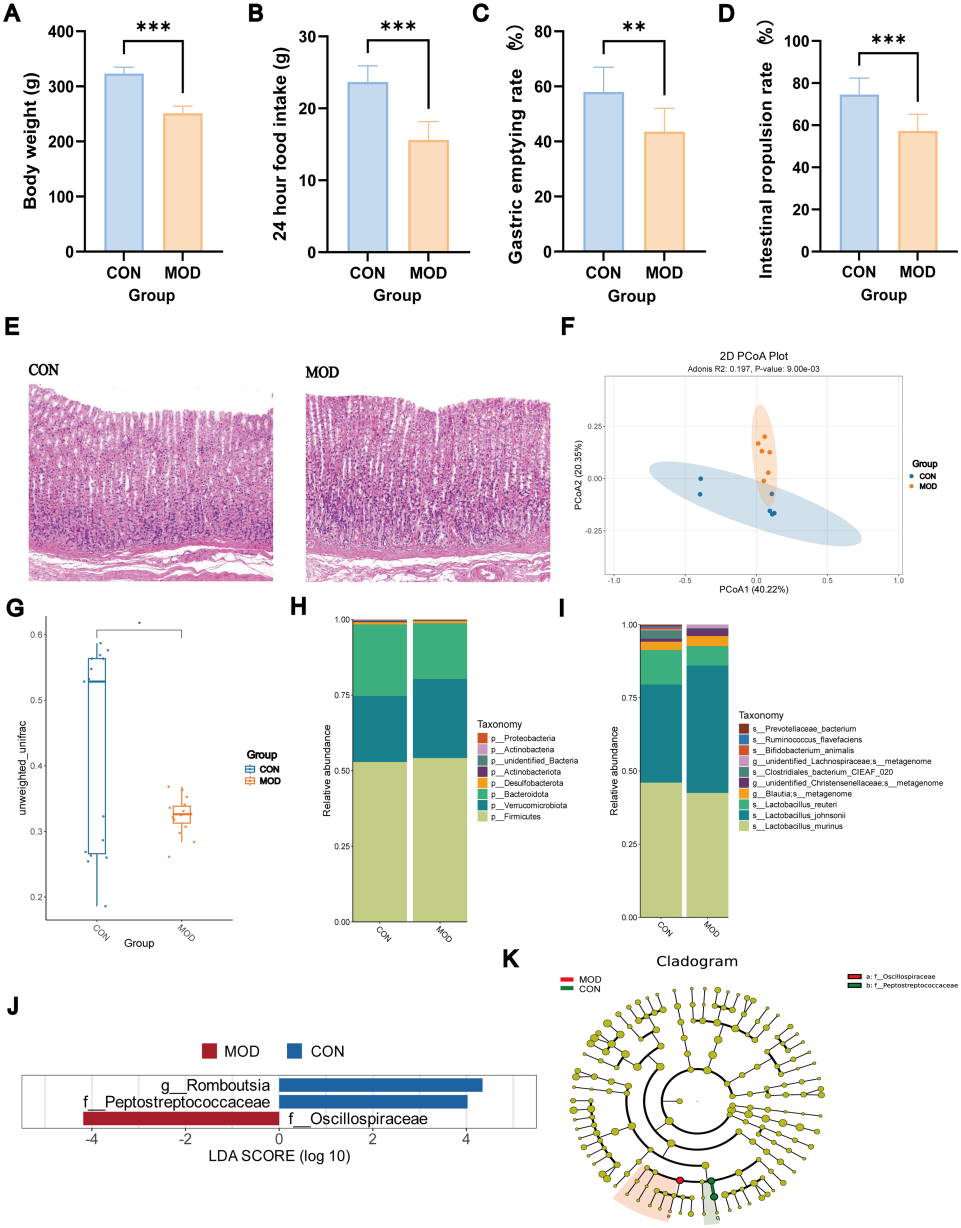
General condition assessment, gastric emptying rate, intestinal propulsion rate, HE staining, and microbial characteristics of rat feces. **(A)** Weight; **(B)** 24 h food intake; **(C)** gastric emptying rate; **(D)** intestinal propulsion rate; (*n* = 10 rats/group), ^**^p < 0.01, ^***^p < 0.001; **(E)** HE staining; **(F)** PCoA analysis; **(G)** unweighted-unifrac analysis; **(H)** differential microbial communities at the phylum level; **(I)** the top 10 differentially expressed microbial communities in each group at the genus level; **(J,K)** LEfSe analysis (*n* = 6 rats/group).

Rats in the MOD group exhibited reduced activity, often huddling together in a curled posture. Their fur became progressively dry, disheveled, and slightly yellowish. Fecal samples were loose, malformed, yellowish-green, and foul-smelling. Compared with the CON group, rats in the MOD group exhibited reduced body weight (*p* < 0.001) and decreased 24-h food intake (*p* < 0.001, Figures 4A,B).

### 3.4 Gastric emptying rate, intestinal propulsion rate, and HE staining

The gastric emptying rate and intestinal propulsion rate were significantly reduced in the MOD group compared with the CON group (*p* < 0.01, *p* < 0.001; Figures 4C,D). HE staining of gastric antrum tissues revealed no significant structural damage, indicating successful induction of the functional dyspepsia model (Figure 4E).

### 3.5 16S rDNA sequencing

Rarefaction and rank abundance curves indicated sufficient sequencing depth and good species evenness (Supplementary Figure 2). Alpha diversity analysis revealed no statistically significant differences between the CON and MOD groups in the ACE, Chao1, Shannon, or Simpson indices (Supplementary Figure 2). In contrast, beta diversity analysis showed clear separation between the two groups in microbial community structure. Significant differences were confirmed by unweighted UniFrac analysis (*p* < 0.05), indicating that FD was associated with alterations in microbial community composition rather than changes in overall species richness or diversity (Figures 4F,G).

At the phylum level, eight differentially abundant bacterial taxa were identified. In contrast to CON group, the MOD group showed increased abundances of *p_Proteobacteria*, *p_Desulfobacterota*, *p_Verrucomicrobiota*, and *p_Firmicutes*, while the abundances of *p_Actinobacteria*, *p_unidentified_Bacteria*, *p_Actinobacteriota*, and *p_Bacteroidota* were decreased (Figure 4H). The top ten differentially abundant species were identified as: *s_Prevotellaceae_bacterium*, *s_Ruminococcus_flavefaciens*, *s_Bifidobacterium_animalis*, *g_unidentified_Lachnospiraceae;s_metagenome*, *s_Clostridiales_bacterium_CIEAF_020*, *g_unidentified_Christensenellaceae;s_metagenome*, *g_Blautia;s_metagenome*, *s_Lactobacillus_reuteri*, *s_Lactobacillus_johnsonii*, and *s_Lactobacillus_murinus* (Figure 4I). However, no significant differences were observed in the relative abundances of *s_Bifidobacterium_adolescentis* and *s_Lachnospiraceae_bacterium_5_1_63FAA* between the CON and MOD groups. Certain bacterial taxa that were substantially different between the two groups were identified by LEfSe (LDA score > 4, Figures 4J,K). By integrating MR and 16S rDNA sequencing results, the abundance of the *p_Actinobacteria* was significantly associated with FD progression. Detailed information on differential microbial features is provided in Supplementary Table 9.

### 3.6 Plasma metabolomics

**Figure 5:**
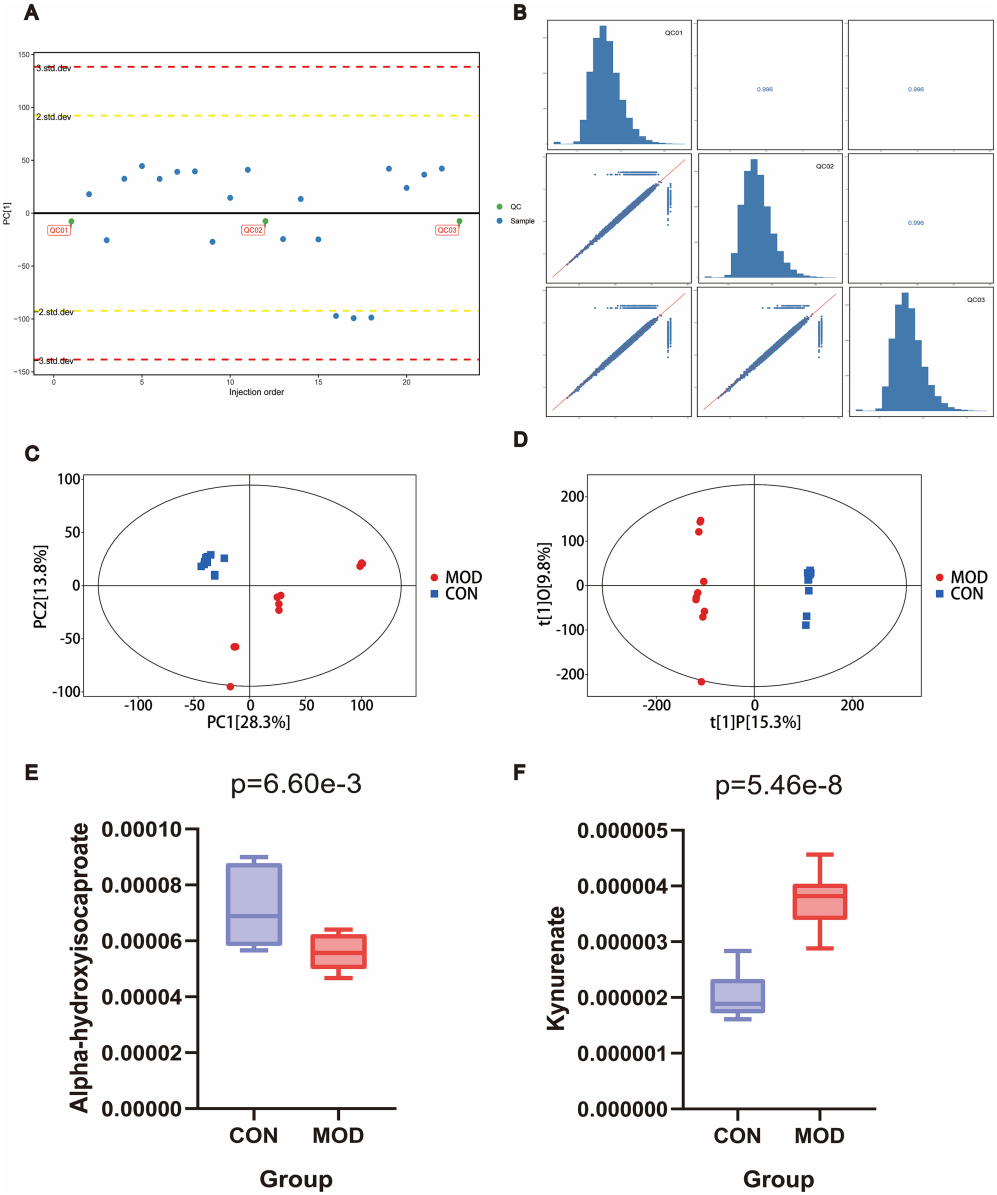
Metabolomics of rat plasma. **(A)** One dimensional distribution map of QC sample PCA-X; **(B)** correlation analysis of QC samples; **(C)** PCA analysis; **(D)** OPLS-DA analysis; **(E)** boxplot plot of alpha-hydroxyisocaproate; **(F)** boxplot plot of kynurenate (*n* = 10 rats/group).

Pooled QC samples, prepared by mixing equal aliquots of all plasma samples, were used for PCA-X analysis to assess analytical stability. The one-dimensional PCA-X score plot showed that all QC samples were located within ±2 STD, and the correlation analysis of QC samples approached 1 (Figures 5A,B), indicating high data quality. Both PCA and OPLS-DA analyses revealed good intra-group clustering and clear separation between groups (Figures 5C,D). There were 1,582 metabolites found to be differently abundant, 951 of which were up-regulated and 631 of which were down-regulated. Integration of MR and metabolomics results indicated that alpha-hydroxyisocaproate and kynurenate were key metabolites involved in the progression of FD (Figures 5E,F). Detailed information on differential metabolites is provided in Supplementary Table 10.

### 3.7 Correlations analysis of differential microbiota and differential metabolites

Spearman correlation analysis revealed a significant positive correlation between *p_Actinobacteria* and alpha-hydroxyisocaproate (*r* = 0.60, *p* = 0.039). However, no significant correlations were observed between *p_Actinobacteria* and kynurenate (*r* = 0.33, *p* = 0.297), or between alpha-hydroxyisocaproate and kynurenate (*r* = −0.49, *p* = 0.106) (Supplementary Figure 3).

## 4 Discussion

Studies have indicated that the pathophysiology of FD is influenced by the gut microbiota and its metabolites (22–24). By focusing on gut microbiota and metabolites, FD progression may be impacted by changes in intestinal immunity and brain function. Our study identified a potential causal association between *p_Actinobacteria* and FD, and further indicated that related metabolites, including kynurenate and alpha-hydroxyisocaproate, may be involved in this relationship. These findings suggest that *p_Actinobacteria* and its associated metabolites could serve as potential biomarkers for FD diagnosis and therapeutic intervention.

We evaluated the possible causal connections between FD and 412 gut microbial characteristics. Three microbial taxa and two microbial composition and activity pathways that are substantially linked to FD were found using MR analysis. Among these, the superpathway of pyridoxal 5′-phosphate biosynthesis and salvage, the *p_Actinobacteria*, and *s_Bifidobacterium adolescentis* were identified as protective factors against FD, whereas the superpathway of menaquinol-8 biosynthesis II and *s_Lachnospiraceae bacterium_5_1_63FAA* were associated with an increased risk of FD. Additionally, no horizontal pleiotropy nor considerable heterogeneity were found, confirming the findings’ robustness. Consistent with the MR results, our 16S rDNA sequencing analysis demonstrated that alpha diversity indices showed no significant differences between groups, indicating that microbial richness and overall diversity were not markedly altered in FD. However, beta diversity analysis revealed significant separation between groups, suggesting that FD, as a functional gastrointestinal disorder, is associated with alterations in microbial community composition rather than overall microbial diversity. These findings indicate that FD is characterized by microbial dysbiosis involving compositional restructuring of the gut microbiota rather than a loss of microbial diversity. Importantly, analysis further confirmed significant alterations in *p_Actinobacteria*, supporting the causal inference derived from MR analysis. By integrating MR and 16S rDNA sequencing results, our findings provide converging evidence that *p_Actinobacteria* plays an important role in FD pathogenesis.

*p_Actinobacteria* can produce secondary metabolites and have been implicated in various functional gastrointestinal disorders (25, 26). According to earlier research, *p_Actinobacteria* abundance in FD models has significantly decreased (27). It inhibits the growth of harmful bacteria by producing short-chain fatty acids (SCFAs) and antimicrobial peptides. Furthermore, through its interaction with intestinal epithelial cells, it prevents harmful substances from entering the bloodstream, regulates the activation of immune cells and the secretion of cytokines, reduces intestinal permeability (28). Our study not only confirmed the decreased abundance of *p_Actinobacteria* in an FD model but also offered genetic proof that *p_Actinobacteria* and FD are causally related. Therefore, we suggest that *p_Actinobacteria* could be a viable treatment target as well as a non-invasive diagnostic biomarker for FD. In addition, two other gut microbial taxa were identified through MR analysis. *Bifidobacterium,* a genus within the *p_Actinobacteria,* is one of the most frequently observed genera in the gastric mucosa of FD (29). By encouraging the synthesis of SCFAs, which give intestinal epithelial cells energy, support the integrity of the intestinal mucosal barrier, and reduce immunological-inflammatory reactions in the duodenal mucosa, it helps maintain intestinal homeostasis. Additionally, *Bifidobacterium* modulates gastrointestinal motility and sensory function via the gut-brain axis, thereby alleviating FD-related symptoms. Clinical studies have confirmed that supplementation with specific strains of *Bifidobacterium* can improve clinical remission rates in FD patients and increase the concentration of SCFAs in both feces and serum (30, 31). *s_Bifidobacterium adolescentis*, an important species within the genus, has an incompletely understood relationship with FD, warranting further investigation through high-quality studies. *s*_*Lachnospiraceae bacterium_5_1_63FAA*, a member of the family *Lachnospiraceae*, was identified in our study as a potential risk factor for FD, showing a positive association with disease development - a finding consistent with previous research (14, 32). However, the precise mechanistic role of *s*_*Lachnospiraceae bacterium_5_1_63FAA* in the progression of FD remains to be elucidated through additional experiments.

At the same time, we identified two pathways reflecting microbial composition and activity that are associated with FD. The active form of vitamin B6, pyridoxal 5′-phosphate, is essential for several enzymatic processes, such as the creation of neurotransmitters and the metabolism of amino acids. The superpathway of pyridoxal 5′-phosphate biosynthesis and salvage encompasses the complete processes of its *de novo* synthesis and recycling from degradation products (33). Our study confirmed that the superpathway of pyridoxal 5′-phosphate biosynthesis and salvage serves as a protective factor against FD. Notably, this pathway is more abundant in the gut microbiota of patients with non-alcoholic fatty liver disease (NAFLD) compared to healthy individuals, despite lower serum levels of pyridoxal 5′-phosphate in NAFLD patients. This phenomenon may be attributed to impaired hepatic metabolism and storage of pyridoxal 5′-phosphate caused by liver disease, or alternatively, to alterations in the gut microbiota that affect the synthesis and utilization of pyridoxal 5′-phosphate. Furthermore, advanced liver conditions such as cirrhosis may disrupt the activity of enzymes involved in vitamin B_6_ metabolism, leading to aberrant pyridoxal 5′-phosphate biosynthesis and salvage (34). These findings further substantiate the beneficial role of pyridoxal 5′-phosphate. Menaquinol-8 is primarily involved in the post-translational modification of proteins that play critical roles in blood coagulation. Recent studies have revealed that the abundance of the superpathway of menaquinol-8 biosynthesis II is positively associated with the risk of breast cancer development (35). Currently, no studies have investigated the correlation between the superpathway of menaquinol-8 biosynthesis II and digestive system disorders. We hypothesize that its activity may modulate metabolic functions of the gut microbiota, thereby potentially impact digestive health.

Furthermore, the absence of reverse causality between these specific microbial taxa and FD supports the hypothesis that gut microbiota dysbiosis may act as a potential trigger for FD, rather than a consequence. This research demonstrated a causal link between specific microbial species and FD, indicating that these microbial markers hold promise not only as auxiliary diagnostic biomarkers but also as potential targets for future microbiome-based interventions of FD.

Subsequently, we also investigated the connection of specific gut microbiota with plasma metabolites. Focusing on the 13 metabolites associated with the *p_Actinobacteria*, integrated metabolomic analysis revealed significant alterations in alpha-hydroxyisocaproate and kynurenate in the FD model, which were closely correlated with *p_Actinobacteria* abundance. Alpha-hydroxyisocaproate, a key intermediate in leucine metabolism, has a favorable correlation with the abundance of beneficial bacteria like *Lactobacillus* and *Bifidobacterium*. Its metabolites are potentially involved in intestinal cellular energy metabolism, influencing energy supply and utilization in gut epithelial cells (36). Additionally, alpha-hydroxyisocaproate exhibits anti-inflammatory properties by modulating the tumor necrosis factor-alpha (TNF-*α*)/interferon-gamma (IFNγ) pathway and inhibiting the synthesis of pro-inflammatory cytokines, such as interleukin-6 and TNF-α (37). Kynurenate is highly expressed in the gastrointestinal lumen (38) and has been demonstrated to suppress intestinal hypermotility and xanthine oxidase activity in models of colonic obstruction (39). In early-stage experimental colitis in rats, kynurenate reduced intestinal motility and suppressed inflammatory activation (40). In the stomach, it exerts protective effects by preserving gastric mucosa, inhibiting gastric acid secretion, and preventing ulcer formation (41, 42). In our study, we observed elevated kynurenate levels in FD rats. Elevated kynurenate levels have also been reported in inflammatory and neuropsychiatric disorders, supporting its association with immune activation and microbiota-related metabolic processes (43, 44). Therefore, the elevated kynurenate observed in FD may be associated with inflammation-related metabolic alterations and host–microbiota interactions, consistent with the microbiota–metabolite associations identified in this study. Our integrated analysis demonstrated that *p_Actinobacteria*, a protective microbial taxon identified by both MR and 16S rDNA sequencing, was associated with FD and may be linked to microbiota–metabolite alterations observed in FD. Although direct evidence linking alpha-hydroxyisocaproate and kynurenate to FD remains limited, their potential associations with gut microbial composition, intestinal motility, barrier function, and immune-inflammatory responses suggest that further investigation into their roles in FD is warranted.

Our study has several limitations. Although MR analysis provided genetic evidence supporting associations between *p_Actinobacteria*, alpha-hydroxyisocaproate, kynurenate, and FD, the animal experiments did not directly manipulate *p_Actinobacteria* abundance or metabolite levels. The observed microbial and metabolic alterations in FD rats therefore represent disease-associated changes rather than direct mechanistic validation of the proposed *p_Actinobacteria*–metabolite–FD pathway. Nevertheless, the concordance between MR findings and animal experimental results provides associative consistency and strengthens the biological plausibility of the involvement of the *p_Actinobacteria*–metabolite axis in FD. Furthermore, the validation of these findings relied solely on animal models and has not yet been confirmed in independent clinical cohorts. Thus, although the MR results were consistent with those from animal experiments, the current evidence remains preliminary. Future studies involving prospective clinical cohorts, *in vitro* functional experiments, and targeted microbiota or metabolite interventions are warranted to further elucidate the mechanistic basis and translational potential of this pathway in FD.

## 5 Conclusion

Our study provides evidence supporting potential associations between specific metabolites, gut microbial taxa, and FD. Through integrated MR and multi-omics analyses, we identified associations between *p_Actinobacteria* and metabolites such as alpha-hydroxyisocaproate and kynurenate and FD, suggesting their possible involvement in the disease process. These findings expand current understanding of the microbial and metabolic features linked to FD and highlight candidate targets for further investigation. Future studies incorporating in vitro functional experiments, longitudinal clinical cohorts, and interventional trials are warranted to clarify the biological roles of these microbial taxa and metabolites in FD and to evaluate their potential utility as biomarkers or therapeutic targets.

## Data Availability

The data sets analyzed in this study are available from the Dutch Microbiome Project (https://dutchmicrobiomeproject.molgeniscloud.org/), the GWAS Catalog (https://www.ebi.ac.uk/gwas/), and the FinnGen consortium (https://www.finngen.fi/en). Summary statistics from GWAS for gut microbiota were sourced from the Dutch Microbiome Project (https://dutchmicrobiomeproject.molgeniscloud.org/). Data on human blood metabolites were acquired from the GWAS Catalog (https://www.ebi.ac.uk/gwas/). Summary statistics for functional dyspepsia were obtained from the FinnGen consortium (https://www.finngen.fi/en). All linkage disequilibrium analyses were performed using the LDlink platform (https://ldlink.nih.gov/?tab=home). Additionally, data from other animal experiments can be obtained from the corresponding author upon reasonable request.

## Footnotes

1
https://r11.finngen.fi/

2
https://ldlink.nih.gov/?tab=home
