# Supplementary material for: The gut microbiota and plasma metabolites on functional dyspepsia: a study integrating Mendelian randomization and experimental validation
Source: Front Med (Lausanne). 2026 Mar 16;13:1793831. doi: 10.3389/fmed.2026.1793831 (PMC13033571; doi:10.3389/fmed.2026.1793831)
Supplement: Supplementary file 1 [file Supplementary_file_1.docx]

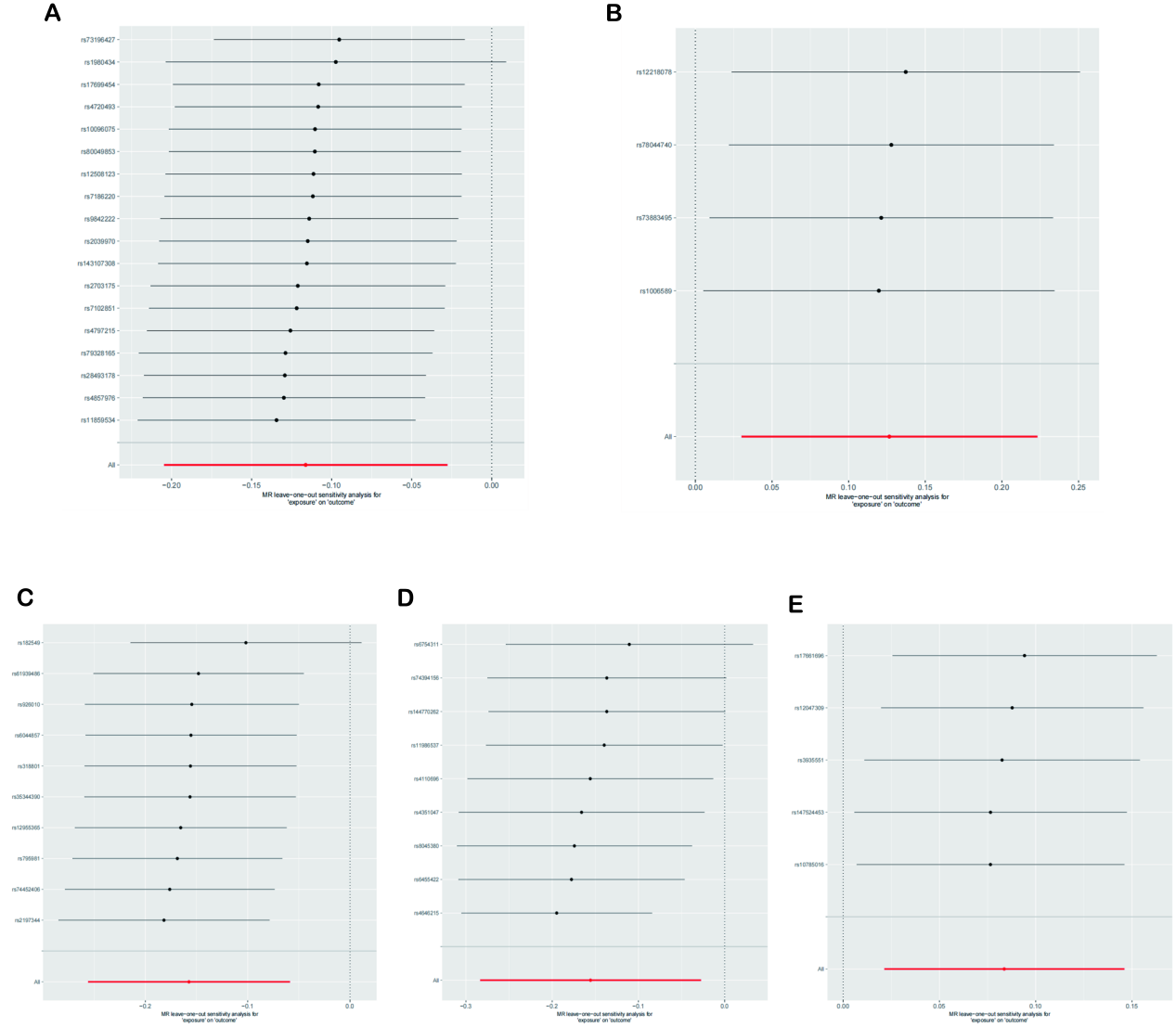


**Supplementary Figure1.** MR leave-one-out sensitivity analysis for gut microbiota on FD. **(A)** Leave-one-out sensitivity analysis of the effect of PWY0-845 superpathway of pyridoxal 5'-phosphate biosynthesis and salvage on FD. **(B)** Leave-one-out sensitivity analysis of the effect of PWY-6263 superpathway of menaquinol-8 biosynthesis II on FD. **(C)** Leave-one-out sensitivity analysis of the effect of k_Bacteria.p_Actinobacteria on FD. **(D)** Leave-one-out sensitivity analysis of the effect of k_Bacteria.p_Actinobacteria.c_Actinobacteria.o_Bifidobacteriales.f_Bifidobacteriacea on FD. **(E)** Leave-one-out sensitivity analysis of the effect of k_Bacteria.p_Firmicutes.c_Clostridia.o_Clostridiales.f_Lachnospiracea on FD.


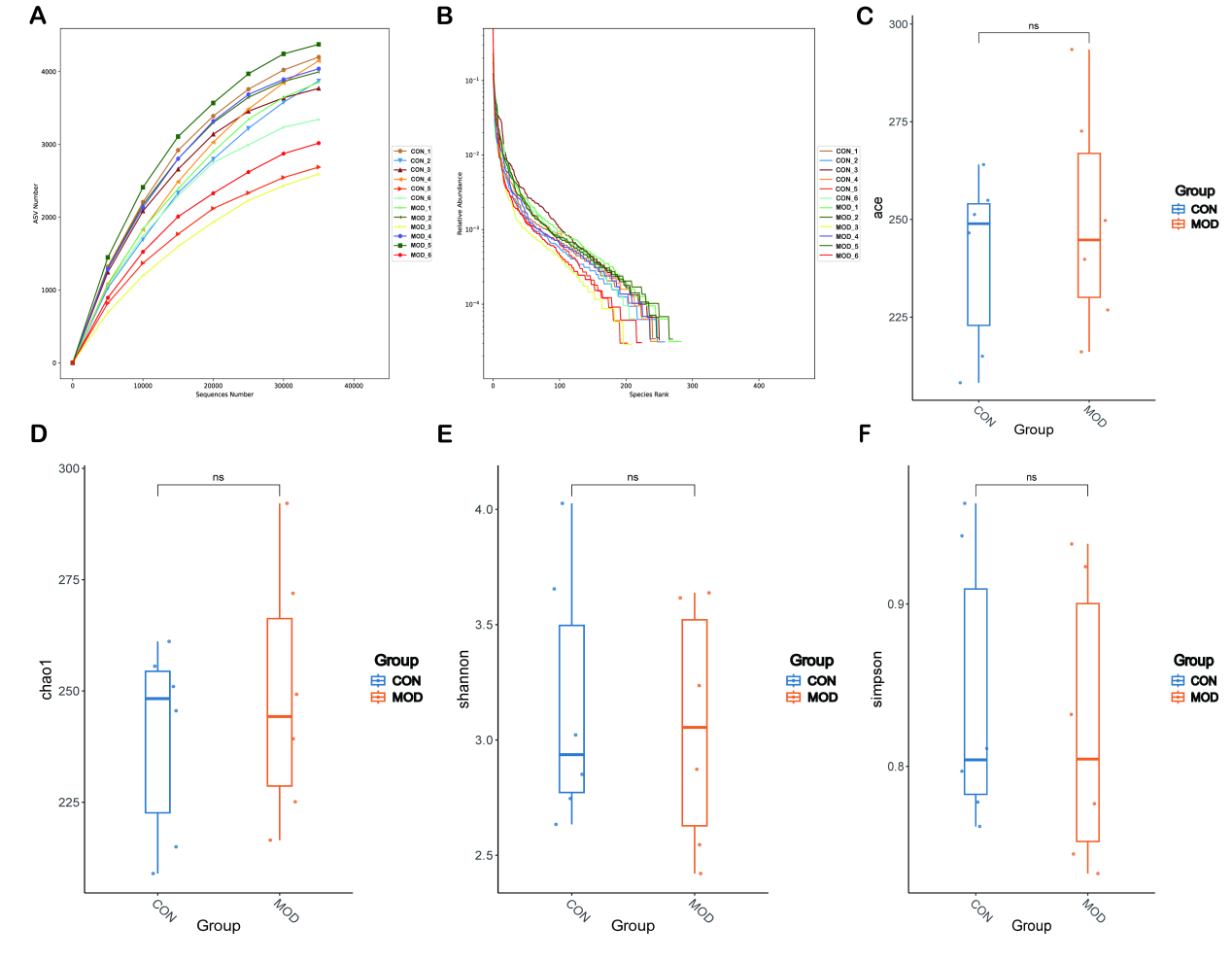


**Supplementary Figure2.** Duodenal microbial analyses. **(A)** rarefaction curve. **(B)** rank-abundance. **(C)** ACE index. **(D)** Chao1 index. **(E)** Shannon index. **(F)** Simpson index.


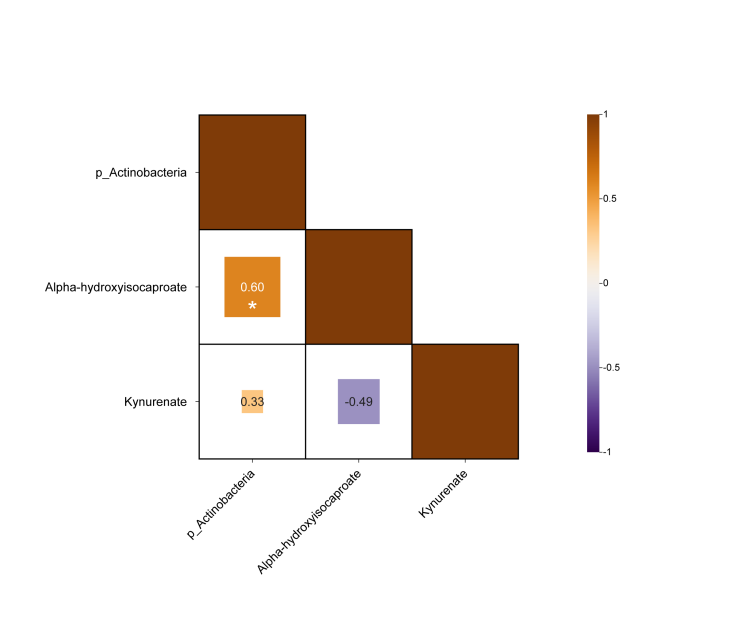


**Supplementary Figure3.** Correlations analysis of differential microbiota and differential metabolites. The numerical values represent the correlation coefficients, *p < 0.05.
